# A Rare Case of Fabry Disease Combined With Idiopathic Multicentric Castleman Disease and Membranous Nephropathy

**DOI:** 10.1016/j.xkme.2026.101401

**Published:** 2026-05-12

**Authors:** Machiko Oka, Rikio Suzuki, Takeshi Akasaka

**Affiliations:** 1 Kidney Disease and Transplant Center, Shonan Kamakura General Hospital, Kamakura, Kanagawa, Japan; 2 Department of Nephrology, Shonan Fujisawa Tokushukai Hospital, Fujisawa, Kanagawa, Japan; 3 Department of Hematology/Oncology, Tokai University School of Medicine, Isehara, Kanagawa, Japan; 4 Department of Cardiology, Shonan Fujisawa Tokushukai Hospital, Fujisawa, Kanagawa, Japan; 5 Kyoundo Hospital, Sasaki Foundation, Chiyoda, Tokyo, Japan

**Keywords:** Fabry disease, multicentric Castleman disease, membranous nephropathy, young female, globotriaosylceramide

## Abstract

The present case documents a 17-year-old woman patient with nephrotic syndrome who was diagnosed with Fabry disease complicated by idiopathic multicentric Castleman disease (iMCD) and membranous nephropathy. Fabry disease, an X-linked lysosomal storage disorder caused by α-galactosidase A deficiency, presents diagnostic challenges in females owing to random X-chromosome inactivation. The presence of zebra bodies in the podocytes, as well as a genetic test, confirmed the diagnosis of Fabry disease, alongside immune complex deposits, which were indicative of membranous nephropathy. In addition, the presence of multiple lymphadenopathies and elevated serum inflammatory marker levels led to the diagnosis of iMCD. Notably, the proteinuria and inflammation rapidly resolved with steroid therapy, suggesting an immune-mediated injury distinct from Fabry-related podocytopathy. This unique case of Fabry disease coexisting with iMCD and membranous nephropathy raises intriguing questions regarding the potential interplay between globotriaosylceramide (Gb3)-induced inflammation and immune dysregulation.

Fabry disease is a rare X-linked genetic disorder caused by mutations in the α-galactosidase A (*GLA*) gene, located at Xq22. This mutation results in the systemic accumulation of globotriaosylceramide (Gb3), which triggers inflammation, fibrosis, and multiorgan damage, particularly affecting the kidneys, heart, and brain.^1^ The estimated prevalence of Fabry disease ranges from 1 in 40,000 to 1 in 170,000, depending on the region.^2^

Castleman disease is a heterogeneous group of lymphoproliferative disorders with diverse histopathological features. Idiopathic multicentric Castleman disease (iMCD) is a rare disorder with an estimated prevalence of 1 to 9 per 1,000,000 inhabitants worldwide.^3^ It is a potentially life-threatening subtype that requires prompt diagnosis and treatment.^4^ The etiology and pathogenesis of iMCD are not fully understood; however, hypotheses involve autoimmunity/autoinflammation, viral or microbial agents, and neoplastic processes.^5^

We hereby present an interesting case of a young female patient diagnosed with both Fabry disease and iMCD. The objective of this case presentation is to further our understanding of the pathological function of Gb3.

### Case Report

A 17-year-old woman exhibited proteinuria and a 10-kg weight reduction over a period of 6 months (height: 160 cm, weight: 56.5 kg). A medical examination conducted 1 year prior had revealed no abnormalities, including no proteinuria. The patient reported experiencing fatigue, bilateral leg edema, and hypotension (99/55 mm Hg) at the time of presentation.

Laboratory investigations revealed the following: serum creatinine, 0.46 mg/dL; serum albumin, 1.5 g/dL; urine protein-to-creatinine ratio, 4.58 g/gCr; hematuria (urine occult blood: 3+); mild anemia (hemoglobin: 10.2 g/dL); white blood cell count, 6,800/μL; platelets, 328,000/μL; serum urea nitrogen, 6.8 mg/dL; and normal liver enzyme levels (aspartate aminotransferase: 10 U/L, alanine aminotransferase: 5 U/L). The patient’s inflammatory markers exhibited elevated levels of C-reactive protein (6.13 mg/dL), interleukin-6 (91 pg/mL), and soluble interleukin-2 receptor (3,357 units/mL). Concurrently, the patient exhibited polyclonal hypergammaglobulinemia characterized by elevated levels of IgG (4,371 mg/dL), IgA (242 mg/dL), and IgM (428 mg/dL). The complement levels (C3: 123 mg/dL, C4: 29.3 mg/dL) were within normal ranges. Antinuclear antibodies exhibited positivity (1:640, homogeneous pattern); however, anti-double-stranded DNA antibodies were negative (Table 1). Consequently, the diagnosis of systemic lupus erythematosus was deemed improbable.

**Table 1:** Laboratory data

Test	Result	Unit
Complete blood count		
WBC	6,800	/μL
Lym	23.4	%
Mono	5	%
Neut	71.1	%
Eo	0.4	%
Baso	0.1	%
Hb	10.2	g/dL
Hct	33.9	%
MCV	76.7	fL
Plt	32.8 × 10^4^	/μL
Biochemical test		
TP	8.5	g/dL
Alb	1.6	g/dL
BUN	6.8	mg/dL
Cr	0.46	mg/dL
UA	3.5	mg/dL
Na	136	mEq/L
K	4	mEq/L
Cl	102	mEq/L
Ca	7.7	mg/dL
IP	3	mg/dL
AST	10	IU/L
ALT	5	IU/L
LDH	114	IU/L
ALP	266	IU/L
γ-GTP	8	IU/L
HbA1c	5.3	%
Ferritin	76.9	ng/mL
CRP	6.13	mg/dL
TSH	2.47	μIU/mL
FT3	1.75	pg/mL
FT4	1	ng/dL
Urinalysis		
pH	7	
Specific gravity	1.011	
Protein	4.58	g/gCr
Occult blood	3+	
Glucose	(-)	
RBC	50-99	/HPF
WBC	1-4	/HPF
Hyaline casts	+	
Epithelial casts	+	
β2MG	425	μg/L
NAG	77.2	U/L
BJP	(-)	
Immunological test		
ESR	≥100	mm/h
IL-6	91	pg/mL
IL-2R	3,357	U/mL
IgG	4,371	mg/dL
IgG4	607	mg/dL
IgA	242	mg/dL
IgM	428	mg/dL
κ light chain	331.6	mg/L
λ light chain	111.5	mg/L
C3	123	mg/dL
C4	29.3	mg/dL
CH50	47	U/mL
RF	(-)	
ANA	640× (homogeneous)	
Anti-DNA antibody	(-)	
Anti-dsDNA antibody	(-)	
Anti-SS-A antibody	(-)	
Anti-SS-B antibody	(-)	
Anti-CCP antibody	(-)	
PR3-ANCA	(-)	
MPO-ANCA	(-)	
Anti-GBM antibody	(-)	
Cryoglobulin	(-)	
Anti-CL/β2GPI antibody	(-)	
Lupus anticoagulant	(-)	
Immunoelectrophoresis	Polyclonal hypergammaglobulinemia type	
M protein	(-)	
Pathogen test		
HBs Ag	(-)	
HCV Ab	(-)	
RPR	(-)	
EBV	(-)	
EBNA	(+)	
HHV-8	(-)	
HIV	(-)	
CMV	(-)	
T-SPOT	(-)	

Abbreviations: CMV, cytomegalovirus; EBV, Epstein–Barr virus; EBNA, Epstein–Barr nuclear antigen; GBM, glomerular basement membrane; HBs Ag, hepatitis B surface antigen; HCV Ab, hepatitis C virus antibody; HIV, human immunodeficiency virus; HHV-8, human herpesvirus 8.

Percutaneous kidney biopsy revealed no significant abnormalities, including foamy podocyte cytoplasm, on hematoxylin-eosin and periodic acid-Schiff staining (Fig 1A). Periodic acid-methenamine-silver staining revealed mild thickening of the glomerular basement membrane with bubbling (Fig 1B). Immunofluorescence staining revealed diffuse granular peripheral deposition of IgG and C3c, and mesangial deposition of IgG, IgA, and IgM (Fig 1C-G). Additionally, autoantibodies, PLA2R, THSD7A, and NELL-1 remained undetected. Electron microscopy revealed electron-dense deposits in the glomerular basement membrane and subepithelial spaces (indicated by the blue arrowhead in Fig 1H), as well as a significant accumulation of zebra bodies in the podocytes with intact foot processes (Fig 1I). These findings indicated a high probability of both membranous nephropathy and Fabry disease.

**Figure 1:**
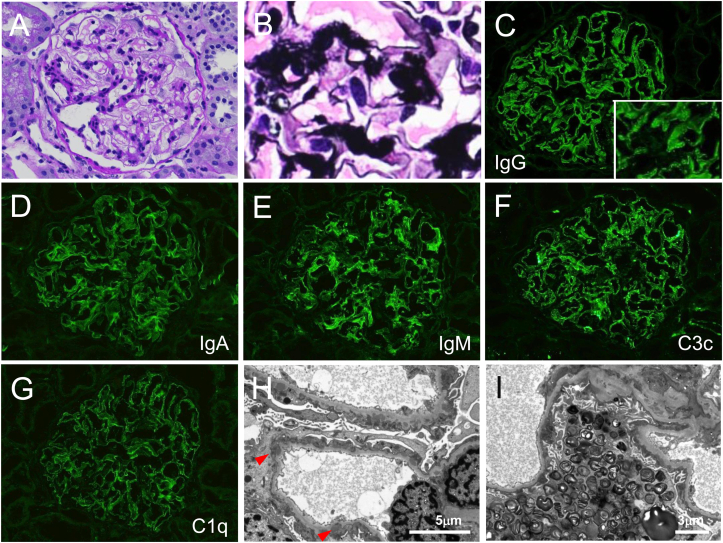
Histopathological phenotypes of kidney biopsy. (A) Periodic acid-Schiff staining of the glomeruli revealed no significant abnormalities (original magnification, ×400). (B) Periodic acid-methenamine silver staining of the glomeruli revealed mild thickening of the glomerular basement membrane with bubbling. (C-G) Immunofluorescence of the glomeruli (original magnification, ×400). (C) IgG staining exhibited a granular pattern in both the basement membrane and the mesangial region. The inset provides a magnified view of the region. (D) IgA is localized in the mesangial region. (E) The presence of nonspecific staining of IgM was observed. (F) C3c staining exhibited a granular pattern in both the basement membrane and the mesangial region. (G) Weak staining of C1q in the mesangial region. (H, I) Electron microscopy of the kidney biopsy. (H) Representative electron-dense deposits in the glomerular basement membrane and subepithelial space as indicated by the red arrowhead (original magnification, ×3,000 scale bar 5 μm). (I) A substantial accumulation of zebra bodies in podocytes exhibiting intact foot processes (original magnification, ×3,000 scale bar 3 μm).

Although GLA enzyme activity was within the normal range (33 mmol/h/mg protein; control range 20-80 mmol/h/mg protein), the serum globotriaosylsphingosine (lyso-Gb3) level was elevated (1.6 mmol/L; control range 0.14-0.75 mmol/L), and urine Gb3 was positive. GLA gene sequencing identified the previously identified pathogenic missense mutation c.593T>C (p.I198T) in exon 4, which is associated with late-onset Fabry disease.^6^ Echocardiography showed normal morphology and left ventricular systolic and diastolic function (left ventricular septal thickness: 8.0 mm, left ventricular posterior wall thickness: 8.0 mm, left ventricular mass index: 71.8 g/m^2^, relative wall thickness: 0.37, left ventricular ejection fraction: 72%, left atrial diameter: 33.0 mm, E/e’: 9.0).

A subsequent ultrasound examination revealed the presence of enlarged superficial lymph nodes in the right supraclavicular fossa (26 mm × 10 mm), bilateral axillae (right: 28 mm × 12 mm; left: 35 mm × 12 mm), and bilateral inguinal regions (right: 28 mm × 7 mm, left: 22 mm × 7 mm). A histological analysis of a left inguinal lymph node revealed angiogenesis and numerous follicles predominantly consisting of CD20^+^ B cells, nonclonal CD10^+^, BCL2^-^, BCL6^+^, and Ki-6^+^ κ-dominant plasma cells within the germinal centers. A bone marrow aspiration revealed no clinically significant abnormalities. These findings, in conjunction with negative human herpesvirus-8 test results, were consistent with the diagnostic criteria for iMCD.^7^

To treat iMCD and membranous nephropathy, 30 mg of prednisolone was administered daily for 3 months. Within 2 weeks, the steroid therapy resulted in a significant reduction in proteinuria (urine protein-to-creatinine ratio decreased to 0.29 g/gCr) and in IL-6 levels (which decreased to 8 pg/mL). As the serum protein loss level diminished, enzyme replacement therapy with agalsidase β (1 mg/kg administered every 2 weeks) was initiated to reduce Gb3 accumulation. At 6 months following the initiation of enzyme replacement therapy, serum lyso-Gb3 levels remained consistently low (less than 0.55 mmol/L; control range 0.14-0.75 mmol/L), and proteinuria did not reemerge. The patient’s weight was restored, and her fatigue resolved.

## Discussion

Diagnosing Fabry disease in female patients presents a substantial clinical challenge owing to random X-chromosome inactivation, which leads to considerable variability in symptoms and GLA activity. The presentation of Fabry disease-related phenotypes in young females is notably less prevalent, often making family histories crucial for identifying at-risk individuals. This case involved a 17-year-old woman who was identified as the proband due to the presence of nephrotic syndrome. She lived in a single-parent household with no paternal information available. Although her mother had no Fabry disease-related phenotypes, paternal inheritance could not be excluded. Alternatively, the identified c.593T>C mutation may have been *de novo*.

The manifestation of kidney damage in Fabry disease, evidenced by proteinuria, becomes apparent around the age of 20 years; however, nephrotic syndrome is extremely rare.^8^ Although a kidney biopsy revealed the presence of zebrabodies caused by Gb3 accumulation, podocyte foot process effacement was not prominent and could not explain the nephrotic syndrome phenotype. However, the presence of immune complex deposits in the glomerular basement membrane, without autoantibodies, suggests coexisting secondary membranous nephropathy. Given the limited mesangial region expansion (Fig 1A) and the weak staining of IgA in the mesangial region (Fig 1D), IgA nephropathy can be excluded as a causative factor for developing membranous nephropathy. In addition, the positive C1q staining in the mesangial region suggests the possibility of lupus nephritis (Fig 1G). Nevertheless, the absence of subendothelial deposition or tubuloreticular inclusions, when considered in conjunction with a negative result for anti-double-stranded DNA antibodies and normal complement levels, does not meet the criteria for lupus nephritis.

In this case, the iMCD combination greatly complicated the pathology. Kidney damage associated with Castleman disease is primarily attributable to amyloidosis and membranoproliferative glomerulonephritis, with membranous nephropathy being exceptionally rare.^9^ The underlying mechanism by which membranous nephropathy manifests is thought to be the result of immune complex formation, precipitated by elevated gamma-globulin levels induced by IL-6. Given that this relatively early (Stage I-II) membranous nephropathy developed concurrently with iMCD and the reduction of IL-6 expression due to steroid treatment was followed by recovery of membranous nephropathy, it was hypothesized that the membranous nephropathy was a sequela of iMCD.

Although iMCD manifests predominantly in middle-aged and older male patients, the patient in this case was a 17-year-old female. The coexistence of 2 exceedingly uncommon diseases in the patient beyond the conventional age range for onset could be explained by the following reasons:

Research studies have demonstrated that GLA-deficient mice exhibited elevated levels of CD68^+^ monocytes in the bone marrow.^10^ Additionally, Gb3 has been observed to stimulate the release of inflammatory cytokines from peripheral mononuclear cells via Toll-like receptor 4.^11^ These findings suggest a potential association between iMCD with autoinflammatory properties, and Fabry disease via Gb3. Although there have been no reports of patients with both diseases concurrently, it has been demonstrated that patients with Fabry disease exhibit chronic inflammation regardless of their genotype.^12^ One notable case study involved a 15-year-old woman patient with Fabry disease who was also diagnosed with anti-neutrophil cytoplasmic antibody-associated vasculitis, which tends to occur after middle age.^13^ This suggests that inflammation induced by Gb3 may have promoted the onset of the disease.

Moreover, studies have demonstrated that Gb3 is associated with aberrant cell proliferation. Fabry disease reportedly increases the risk of certain malignancies, such as urinary tract cancer and melanoma.^14^^,^^15^ In addition to blood cancers, such as Burkitt's lymphoma, elevated Gb3 levels have been observed in cases of breast, colon, pancreatic, and renal cancers.^16^^,^^17^ Although Gb3 is not necessarily elevated in young heterozygous patients, Lyso-Gb3 is significantly elevated. It has been suggested that Lyso-Gb3 exerts a highly pathogenic effect on the myocardium and endothelial cells and induces oxidative DNA damage in kidney cells.^18^^,^^19^ Additionally, enzyme replacement therapy using agalsidase β has been observed to increase the proportion of natural killer cells in patients with Fabry disease, suggesting a potential link between Fabry disease and oncogenesis.^20^

Even though the interactions between these 2 rare diseases remain unclear, the accumulation of oncogenic Gb3/Lyso-Gb3 caused by Fabry disease in lymphocytes could lead to chronic inflammation and cellular damage, resulting in the development of iMCD. The patient’s clinical course should be meticulously monitored for any potential subsequent malignancies. Further research is essential to investigate the potential association between these rare disorders.
